# 582. Risk Factors for Progression to Hospitalization in Adolescents Presenting with Mild or Moderate COVID-19

**DOI:** 10.1093/ofid/ofab466.780

**Published:** 2021-12-04

**Authors:** Melanie Dubois, Jeffrey Campbell, Gabriella S Lamb, Gabriella S Lamb, Mari M Nakamura

**Affiliations:** 1 Boston Children's Hospital, Harvard Medical School, Boston, MA; 2 Boston Children's Hospital, Boston, MA; 3 Boston Children's Hospital, Harvard medical school, Jamaica Plain, MA

## Abstract

**Background:**

Most adolescents (age 12-17 years) with COVID-19 have mild disease. However, certain comorbidities may be risk factors for disease progression, and hospitalization rates for this age group have increased. Adolescents and adults with mild to moderate COVID-19 are eligible for monoclonal antibody therapy. To identify subgroups likely to benefit from this intervention, we evaluated the relationship between comorbidities and need for hospitalization in US adolescents presenting with mild to moderate COVID-19.

**Methods:**

We analyzed presence of comorbidities and need for hospitalization within 28 days of diagnosis for adolescents in the PIDTRAN registry, a multicenter retrospective cohort of US pediatric patients with COVID-19. Comorbidities assessed included obesity, chronic kidney disease (CKD), diabetes (DM), immunosuppressive disease or treatment (IS), sickle cell disease (SCD), congenital/acquired heart disease (CHD), neurologic disease/neurodevelopmental disorders (ND), medical-related technology dependence (MTD), and pulmonary disease requiring daily inhaled corticosteroids (PD). We used multivariable logistic regression to determine race/ethnicity-adjusted associations between comorbidities and hospitalization.

**Results:**

1574 patients met inclusion criteria, of whom 180 (11.4%) were hospitalized within 28 days of COVID-19 diagnosis. In a race/ethnicity-adjusted model, the following comorbidities were independently associated with increased odds of hospitalization: IS (OR 10.8 [95%CI 5.4 – 21.7]); CKD (OR 5.1 [95%CI 1.0 – 25.6]); DM (OR 4.2 [95%CI 1.7 – 10.6]); SCD (OR 3.4 [95%CI 1.1 – 10.6]). ND (OR 3.0 [95%CI 1.7 – 5.4]); and obesity (OR 2.0 [95%CI 1.1 – 3.9]). Notably, CHD, MTD, and PD were not independently associated with hospitalization. There was no effect modification of race/ethnicity on the association between obesity or DM and hospitalization.

Table 1: Characteristics of adolescents in our cohort

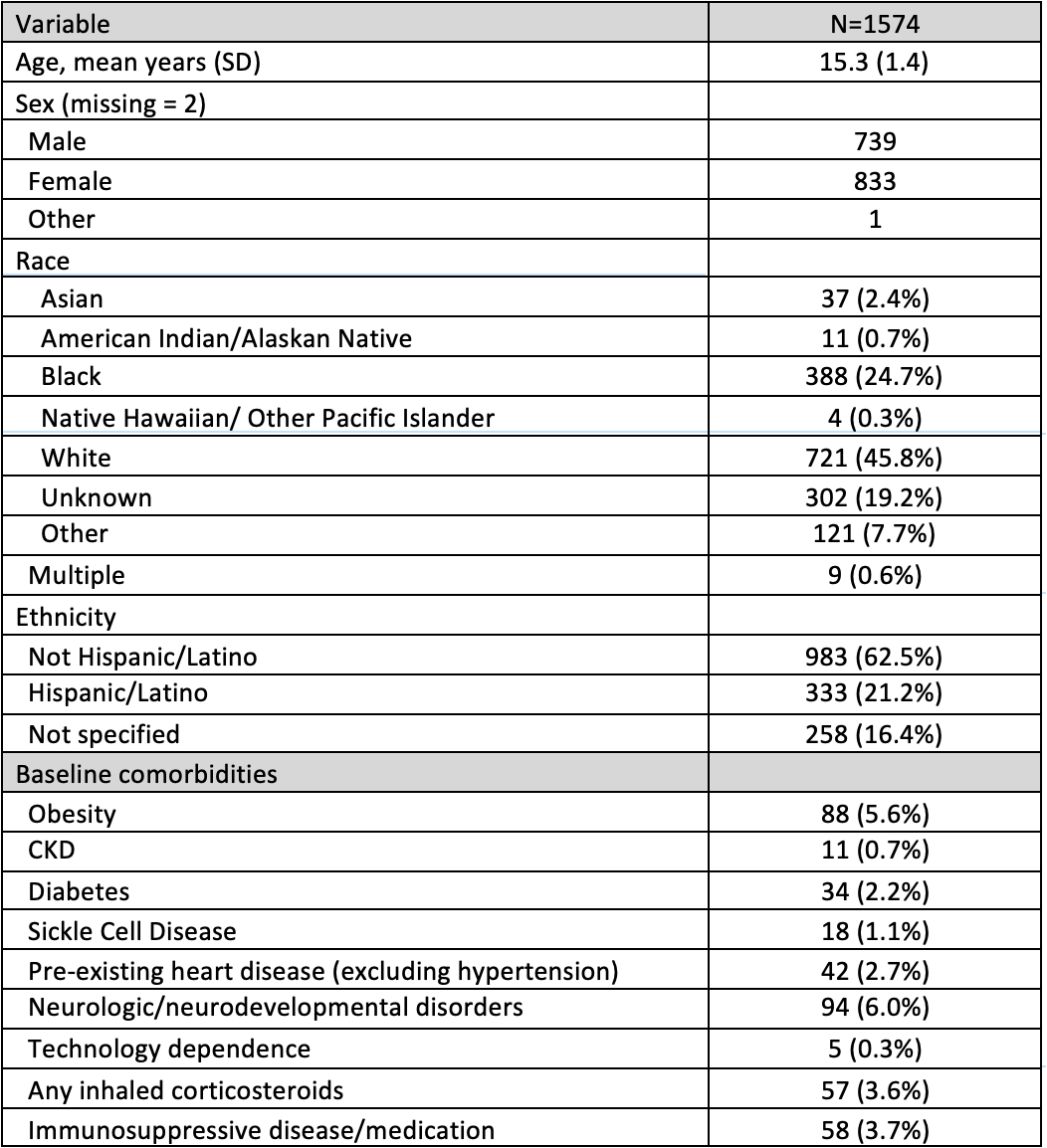

Figure 1. Association between comorbidities and hospitalization. Model 1: comorbidities only. Model 2: comorbidities, adjusted for race/ethnicity. Abbreviations: CKD – chronic kidney disease; SCD – sickle cell disease; ICS – inhaled corticosteroids.

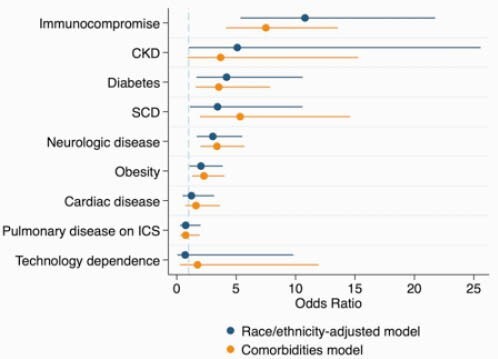

**Conclusion:**

IS, CKD, DM, SCD, ND, and obesity were associated with increased odds of hospitalization in adolescents presenting with mild to moderate COVID-19. Adolescents with these comorbidities should be prioritized for consideration of treatment with monoclonal antibodies.

**Disclosures:**

**Gabriella S. Lamb, MD, MPH**, Nothing to disclose

